# Complete Genome Sequence of Pseudomonas taiwanensis WRS8, a Plant Growth-Promoting and Biotite-Weathering Strain

**DOI:** 10.1128/MRA.00252-21

**Published:** 2021-05-06

**Authors:** Cheng Cheng, Qi Wang, Xuewei Li, Yuanyuan Liu

**Affiliations:** a Key Laboratory of Agrometeorology of Jiangsu Province, Department of Ecology, School of Applied Meteorology, Nanjing University of Information Science and Technology, Nanjing, China; University of Arizona

## Abstract

Pseudomonas taiwanensis WRS8 is a biotite-weathering organism isolated from wheat root. Here, we report its complete genome sequence, which may shed light on its role in plant growth promotion and biotite weathering.

## ANNOUNCEMENT

It is widely assumed that silicate mineral weathering plays an important role in soil formation, inorganic nutrient availability, and the protection of stone cultural relics and architecture (1). Numerous studies have documented that microorganisms can significantly accelerate mineral weathering though acidolysis, chelation, or oxidoreduction reactions (2). Pseudomonas taiwanensis WRS8, a plant growth-promoting and biotite-weathering bacterial strain, was isolated from the roots of wheat (Triticum aestivum L. Yangmai-13) in Qixia District, Nanjing City, Jiangsu Province, China (32°9′N, 118°57′E) according to the method described by Sun et al. (3). Briefly, 0.2 mg root was washed with deionized water, sterilized by sequential immersion in 75% (vol/vol) ethanol for 2 min and 1% mercuric chloride for 1 min, and washed three times with sterile water to remove surface sterilization agents. Then, it was ground and serially diluted, and the dilutions were spread onto plates containing LB medium. After 5 days of incubation, colonies were picked and isolated. Based on analyses of the cellular morphology, the bacterial partial 16S rRNA gene sequence similarity, and the *gyrB* and *rpoD* gene sequences, strain WRS8 was identified as one of the Pseudomonas taiwanensis species (4).

After 2 passages of propagation, strain WRS8 was grown on LB medium for 2 days. A single colony was picked for DNA extraction using a bacterial DNA kit (OMEGA) according to the manufacturer’s instructions. Then, it was quantified using a TBS-380 fluorometer (Turner BioSystems, Inc., Sunnyvale, CA). A DNA sample with high quality (optical density at 260/280 nm [OD_260/280_] = 1.8∼2.0, >6 μg) was utilized to construct a fragment library and then sequenced on both the Pacific Biosciences RS II platform (Shanghai Biozeron Co., Ltd.) and the Illumina HiSeq platform (PE150 mode, Shanghai Biozeron Co., Ltd.). For the PacBio sequencing, a library was prepared using the SMRTbell template prep kit v. 1.0, and fragments smaller than 20 kb were removed with the automated BluePippin size selection system (Sage Science, Beverly, MA). A total of 16,641,890 long reads and 2,496,283,500 bp were produced. For Illumina paired-end sequencing, 3 μg genomic DNA was used for sequencing library construction, and paired-end libraries with insert sizes of ∼400 bp were prepared. A total of 2,496 Mb raw data of 450 bp were obtained.

The PacBio sequencing reads were binned and filtered by quality (Q score, >10) with Barapost v. 2020-06-26 (5), which yielded a total of 3,802,278,545 bp distributed in 285,036 reads with an *N*_50_ value of 16,695 bp. The prepared reads were assembled into 1 contig using Canu v. 2.0 (https://github.com/marbl/canu) (6). The contig was checked for circularization using the Tablet v. 1.21.02.08 software (7) and specified through manual inspection for overlapping ends. As for the Illumina reads, after subread filtering with Trimmomatic v. 0.36, 2,257 Mb high-quality paired-end reads were generated with a >100-fold depth of coverage. Then, these Illumina reads were used to correct the PacBio errors using Bowtie 2 v. 2.3.5.1 (8) and Pilon v. 1.23 (9) software. Default parameters were used for all software.

The Pseudomonas taiwanensis WRS8 genome consists of a 5,851,681-bp circular chromosome (GC content, 61.84%). In addition, the genome sequence was rotated using the Unicycler v. 0.4.8+dfsg-2build1 pipeline. A total of 5,244 protein-coding genes with an average length of 985 kb, 22 rRNAs, and 75 tRNA genes were found in this genome.

### Data availability.

The assembled complete genome sequence of strain Pseudomonas taiwanensis WRS8 has been deposited at GenBank under accession number CP062699. The raw reads were deposited in the Sequence Read Archive under accession number SRR12825693 for the PacBio reads and SRR12825694 for the Illumina reads under BioProject accession number PRJNA668451 and BioSample number SAMN16409531.
